# Comparison of two different measurement methods in evaluating basilar atherosclerotic plaque using high-resolution MRI at 3 tesla

**DOI:** 10.1186/s12880-018-0293-1

**Published:** 2018-12-03

**Authors:** Luguang Chen, Qian Zhan, Wenjia Peng, Tao Song, Qi Liu, Jianping Lu

**Affiliations:** Department of Radiology, Changhai Hospital of Shanghai, The Second Military Medical University, No.168 Changhai Road, Shanghai, 200433 China

**Keywords:** Basilar artery, Atherosclerosis, Magnetic resonance imaging

## Abstract

**Background:**

To compare the Self-referenced and Referenced measurement methods in assessing basilar artery (BA) atherosclerotic plaque employing dark blood high-resolution MRI at 3 Tesla.

**Methods:**

Forty patients with > 20% stenosis as identified by conventional MRA were recruited and evaluated on a 3 Tesla MRI system. The outer wall, inner wall and lumen areas of maximal lumen narrowing site and the outer wall and lumen areas of sites that were proximal and distal to the maximal lumen narrowing site were manually traced. Plaque area (PA), stenosis rate (SR) and percent plaque burden (PPB) were calculated using the Self-referenced and Referenced measurement methods, respectively. To assess intra-observer reproducibility, BA plaque was measured twice with a 2-week interval in between measurements.

**Results:**

Thirty-seven patients were included in the final analysis. There were no significant differences in PA, SR and PPB measurements between the two methods. The intra-class coefficients and coefficient of variations (CV) ranged from 0.976 to 0.990 and from 3.73 to 5.61% for the Self-referenced method and ranged from 0.928 to 0.971 and from 4.64 to 9.95% for the Referenced method, respectively. Both methods are effective in the evaluation of BA plaque. However, the CVs of the Self-referenced method is lower than the Referenced measurement method. Moreover, Bland-Altman plots showed that the Self-referenced method has a narrower interval than the Referenced measurement method.

**Conclusions:**

The Self-referenced method is better and more convenient for evaluating BA plaque, and it may serve as a promising method for evaluation of basilar atherosclerotic plaque.

## Background

Atherosclerosis is a disease that progresses slowly and silently over decades, and the slow progress offers a chance for diagnosis before symptoms occur [1, 2]. Intracranial atherosclerosis is the most common reason for mortality in Asian populations [3]. The basilar artery (BA) is one of the largest intracranial arteries and is located in the posterior cerebral circulation. Basilar atherosclerotic plaque usually occurs in patients with ischaemic stroke and transient ischaemic attack (TIA) [4]. Evaluation of morphologic characteristics of BA plaque (such as PA, plaque area; SR, stenosis rate and PPB, percent plaque burden) is important and may guide treatment decisions in the clinical setting.

**Fig. 1:**
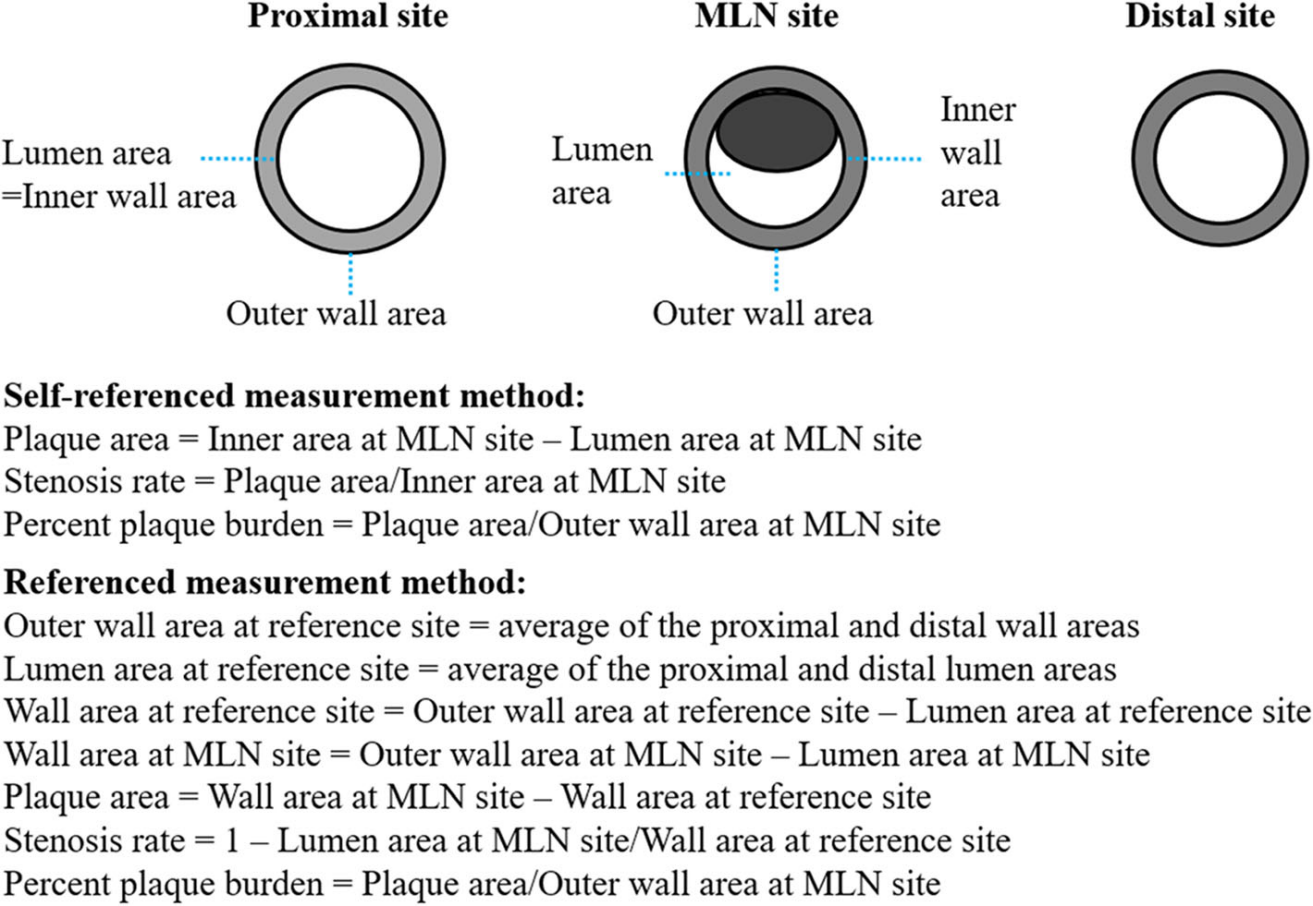
Definitions of the Self-referenced and Referenced measurement methods and their calculation rules for assessing BA plaque

The rapid development of magnetic resonance imaging technology, especially at 3 Tesla field strength, offers a significant improvement in the signal-to-noise ratio, vessel wall to lumen contrast-to-noise ratio and image quality compared to imaging at 1.5 Tesla [5]. Dark blood high-resolution magnetic resonance imaging (HRMRI) has been demonstrated as a non-invasive and useful technique for evaluating the vessel wall in in vivo atherosclerotic disease [1]. It has been used to assess various major arteries in the whole body including the intracranial [6–10], carotid [11–15], coronary [16–18] and peripheral arteries [19–21] and the aorta [11, 22]. Accurate and fast assessment of the plaque burden and morphology of the basilar artery are paramount for determining treatment strategies for patients. Kim et al. used the maximal lumen narrowing (MLN) sites as the referenced sites to evaluate the degree of stenosis in basilar atherosclerotic plaque using HRMRI [23]. Previous studies have used the nearest plaque-free or minimally diseased segments proximal and distal to the MLN sites as the referenced sites to calculate morphologic parameters (herein referred to as the Referenced measurement method) [24–26]. In the present study, we have also employed the MLN sites as the referenced sites to compute the morphological parameters (herein referred to as the Self-referenced measurement method). Figure 1 shows the definitions of the Self-referenced and Referenced measurement methods and their calculation rules for assessing BA plaque. However, studies on the comparison of the Self-referenced and Referenced measurement methods in assessing basilar atherosclerotic plaque have been limited. We hypothesized that the Self-referenced measurement method is better than the Referenced measurement method in evaluating plaque morphologic characterization. To the best of our knowledge, no such studies have been reported yet except our group [27].

Therefore, the goal of this study was to compare the Self-referenced and Referenced measurement methods in assessing basilar atherosclerotic plaque employing dark blood HRMRI at 3 Tesla.

## Methods

### Subjects

This prospective study was approved by the Committee on Ethics of Biomedical Research, Changhai Hospital of Shanghai, and written informed consent was obtained from each patient. Forty patients with > 20% stenosis as identified by conventional contrast-enhanced magnetic resonance angiography were recruited during the period from January 2014 to July 2016. The inclusion criteria were listed as follows: 1) Patients who had ischaemic stroke or TIA in the basilar artery territory within one month; 2) Occurrence of an ischaemic event in a vascular area that lies outside of the stenotic basilar artery; and 3) More than two risk factors for cardiovascular disease (e.g., hypertension, diabetes, smoking and hypercholesterolemia). The following criteria were used to exclude patients: 1) the degree of stenosis of the basilar artery was normal, occluded or < 20%; 2) arteritis; 3) claustrophobia; and 4) poor image quality.

### Magnetic resonance imaging

Cross-sectional imaging was performed on a 3 Tesla MRI system (MAGNETOM Skyra, Siemens medical solution, Erlangen, Germany) using a standard 20-channel phased-array head/neck coil, with a peak gradient strength of 45 mT/m and a slew rate of 200 mTm^− 1^ ms^− 1^. Three-dimensional time-of-flight magnetic resonance angiography (3D TOF-MRA) images were used for HRMRI image positioning and obtained with repetition time/echo time (TR/TE) = 21/3.4 ms, field of view (FOV) = 180 × 200 mm^2^, matrix = 330 × 384, thickness = 0.7 mm, and average = 1, acquisition time (TA) = 4 min 40 s. The main parameters of two-dimensional T2-weighted turbo spin echo (T2W TSE) were TR/TE = 2890/46 ms, FOV = 100 × 100 mm^2^, matrix = 256 × 256, thickness = 2 mm, Gap = 0.5 mm, echo train length = 20, and averages = 2, TA = 3 min 40 s. Fat saturation was applied to suppress signal from adjacent fatty tissues and improve identification of vessel wall boundaries. The black blood method with a regional saturation pulse of 60 mm thickness was employed to saturate the inflow arterial signal. The total scan time was approximately 8 min.

### Image analysis

**Fig. 2:**
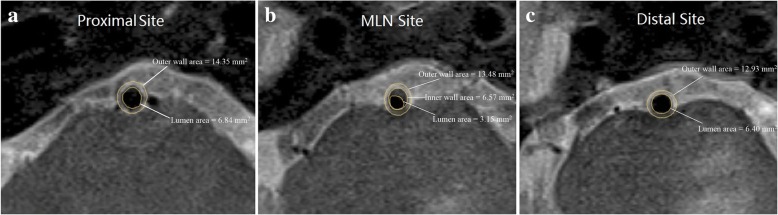
Examples of outer wall areas, inner wall areas and lumen areas at the proximal sites (**a**), maximal lumen narrowing (MLN) sites (**b**) and distal sites (**c**) that were traced manually in a 53-year-old female patient with basilar atherosclerotic plaque. For the Self-referenced measurement method, the plaque area is 3.42 mm^2^, stenosis rate is 0.521, and percent plaque burden is 0.254. For the Referenced measurement method, the outer wall area at the reference site is 13.64 mm^2^, the lumen area at reference site is 6.62 mm^2^, the wall area at the reference site is 7.02 mm^2^, the wall area at the MLN site is 10.33 mm^2^, the plaque area is 3.31 mm^2^, the stenosis rate is 0.551, and the percent plaque burden is 0.246

Two experienced radiologists with 5 and 6 years of experience in vessel wall imaging and who were blinded to the clinical information of each patient assessed the image quality by consensus using 4-scale scores (score 1, poor quality; score 2, adequate quality; score 3, good quality; score 4, excellent quality) [28]. Images with a score of 1 were excluded from the final analysis. Quantitative measurement was carried out on images with a score of ≥2. The outer wall, inner wall and lumen areas of the MLN site and the outer wall and lumen areas of the proximal and distal sites were manually traced using advanced image software (CMRTools, Cardiovascular Imaging Solutions, London, UK; Fig. 2). The calculation of PA, SR and PPB using the Referenced measurement method was carried out as per a previous study [24], which used the nearest normal segments proximal and distal to the MLN sites as the reference to calculate the above parameters (Fig. 1). In brief, outer wall and lumen areas at the reference points were averages of the proximal and distal outer wall and lumen areas. Wall area was calculated by subtracting the lumen area from the outer wall area. The calculations for PA, SR and PPB were as follows: PA = wall area at MLN site – wall area at the reference site, SR = 1-lm area at MLN site/lumen area at the reference site, PPB = PA/outer wall area at MLN site. For the Self-referenced measurement method, the MLN site was used to as the reference. PA = inner wall area at MLN site - lumen area at MLN site, SR = PA/ inner wall area at MLN site, PPB = PA/outer wall area at MLN site (Fig. 1). The distance between proximal and distal reference locations was calculated and recorded (*distance* = m * Slice thickness + (m - 1) * Gap*, m* indicates the number of slices between both locations). To measure the intra-observer variability, BA plaque was measured twice at two different time points that were separated by a 2-week interval to avoid any recall bias.

### Statistical analysis

All statistical analyses were performed using the SPSS software for Windows (version 16.0, SPSS Inc., Chicago, IL, USA) and MedCalc (version 13.0.0.0, MedCalc Software, Mariakerke, Belgium). A Kolmogorov-Smirnov test was used to test for normal distribution. Quantitative data were described as the means ± standard deviation. Qualitative data were expressed as count (percentage). Statistically significant differences between the Self-referenced and Referenced measurement methods and between the repeated measurements of PA, SR and PPB with both methods were assessed using a two-tailed paired t-test. The coefficient of variation (CV) was calculated by determining the standard deviation (SD) of the two paired measurements of T2W images and dividing the mean of those measurements. The intraclass correlation coefficient (ICC) with a two-way mixed model and single consistency type was determined and used to evaluate the agreement between the repeat measurements. According to Shout and Fleiss [29], values of ICC < 0.4 represent poor agreement, 0.4 to 0.75 represent good agreement, and > 0.75 represent excellent agreement. Bland-Altman plots were also derived for those measurements [30] and bias and limits of agreement were calculated. A *P*-value of < 0.05 was considered as statistically significant.

## Results

While all patients completed HRMRI examinations, three patients were excluded in the final analysis because of poor image quality. The scores of images quality were as follows: 2 in 5 patients, 3 in 21 patients and 4 in 11 patients. Thus, thirty-seven patients (28 males and 9 females, 47–79 years old, mean age = 62 years) were included in the study, and quantitative analysis was performed with their data. The patient demographics were presented in Table 1. The time interval between the stroke event and plaque imaging was also recorded, and the average time was 16.2 ± 8.2 days. The distances between proximal and distal reference locations were 11.12 ± 3.78 mm.

**Table 1:** Patient demographics

Variable	*n* = 37
Male sex	28 (75.7%)
Age (years)	62.4 ± 10.1
Body mass index	24.4 ± 3.1
Hypertension	27 (73.0%)
Systolic pressure(mmHg)	159.0 ± 26.5
Diastolic pressure (mmHg)	94.8 ± 17.0
Diabetes mellitus	17 (45.9%)
Fasting blood-glucose (mg/dL)	8.3 ± 9.7
Hyperlipidaemia	19 (51.4%)
Total cholesterol (mmol/L)	4.08 ± 1.05
Triglyceride (mmol/L)	1.41 ± 0.53
High-density lipoprotein (mmol/L)	1.06 ± 0.19
Low density lipoprotein (mmol/L)	2.52 ± 0.95
Stenosis rate	0.69 ± 0.18
Apolipoprotein A1 (g/L)	1.14 ± 0.23
Apolipoprotein B (g/L)	0.83 ± 0.19
Smoking	16 (43.2%)

### Comparison of self-referenced and referenced measurement methods

**Fig. 3:**
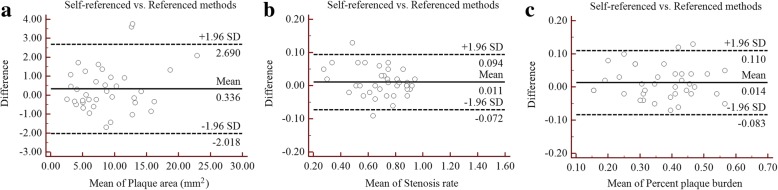
Comparison of the Self-referenced and Referenced measurement methods for BA plaque using Bland-Altman plots, plaque area (**a**), stenosis rate (**b**) and percent plaque burden (**c**)

The results of the Self-referenced and Referenced measurement methods are presented in Table 2. PA determined by both measurement methods were 9.015 ± 4.916 mm^2^ and 8.678 ± 4.634 mm^2^, respectively. The SR values were 0.699 ± 0.172 and 0.688 ± 0.184 for the Self-referenced and Referenced methods, respectively. In addition, the PPB values of the two measurement methods were 0.375 ± 0.111 and 0.361 ± 0.117, respectively. The bias and limit of agreement measurements using Bland-Altman plots for the PA, SR and PPB were 0.336 (− 2.018, 2.690), 0.011 (− 0.072, 0.094) and 0.014 (− 0.083, 0.110) (Fig. 3), respectively. There were no statistically significant differences between the two methods in those measurements (*p*-values > 0.05).

**Table 2:** Comparison of Self-referenced and Referenced measurement methods in assessing basilar plaque

	Self-referenced (Mean ± SD)	Referenced (Mean ± SD)	Bias	LoA	*p*
Plaque area (mm^2^)	9.015 ± 4.916	8.678 ± 4.634	0.336	(−2.018, 2.690)	0.097
Stenosis rate	0.699 ± 0.172	0.688 ± 0.184	0.011	(−0.072, 0.094)	0.121
Percent plaque burden	0.375 ± 0.111	0.361 ± 0.117	0.014	(−0.083, 0.110)	0.761

*SD* Standard deviation, *LoA* Limit of Agreement

### Reproducibility of the self-referenced measurement method

**Fig. 4:**
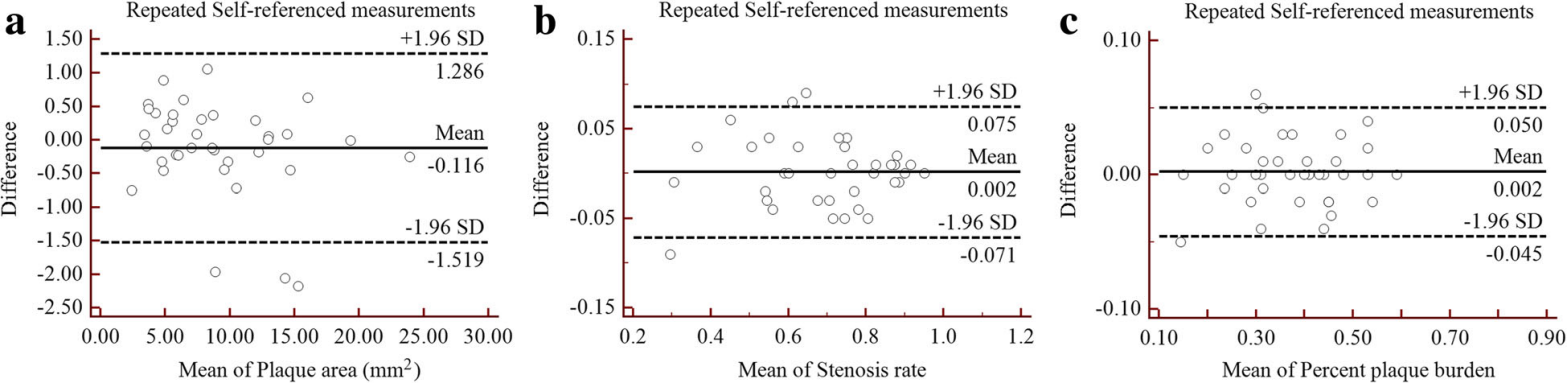
Bland-Altman plots of reproducibility for the Self-referenced method, plaque area (**a**), stenosis rate (**b**) and percent plaque burden (**c**) of BA plaques were evaluated using the Self-referenced measurement method

The results for the Self-referenced measurement reproducibility analysis are presented in Table 3. Repeated measurements with the Self-referenced method were 8.954 ± 4.833 mm^2^ and 9.071 ± 5.023 mm^2^ for PA, 0.697 ± 0.173 and 0.695 ± 0.172 for SR, and 0.374 ± 0.112 and 0.372 ± 0.112 for PPB. No statistically significant differences were observed for those measurements (*p*-values > 0.05). The results indicate an excellent reproducibility for the repeated measurements, with the ICC and CV values ranging from 0.976 to 0.990 and 3.73 to 5.61%, respectively. Excellent agreement was also observed in the repeated measurement reproducibility analysis employing Bland-Altman plots (Fig. 4).

**Table 3:** Reproducibility of Self-referenced measurement method in assessing basilar plaque

	Measurement 1 (Mean ± SD)	Measurement 2 (Mean ± SD)	ICC (95% CI)	CV (%)	*p*
Plaque area (mm^2^)	8.954 ± 4.833	9.071 ± 5.023	0.990 (0.981–0.995)	5.61	0.329
Stenosis rate	0.697 ± 0.173	0.695 ± 0.172	0.977 (0.955–0.988)	3.73	0.759
Percent plaque burden	0.374 ± 0.112	0.372 ± 0.112	0.976 (0.954–0.988)	4.59	0.549

*SD* Standard deviation, *ICC* Intra-class coefficient, *CI* Confidence interval, *CV* Coefficient of variability

### Reproducibility of the referenced measurement method

**Fig. 5:**
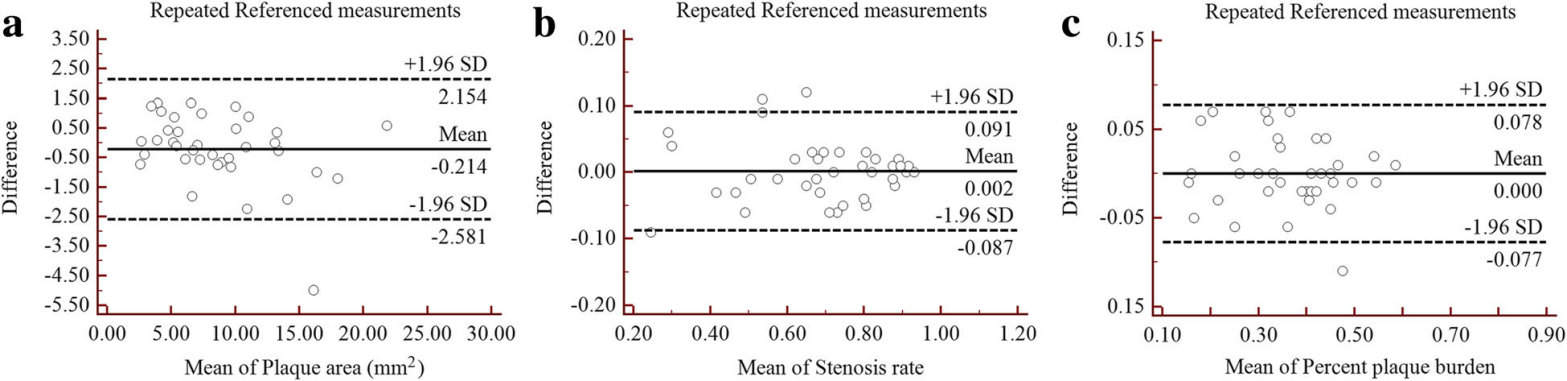
Bland-Altman plots of reproducibility for the Referenced methods, plaque area (**a**), stenosis rate (**b**) and percent plaque burden (**c**) of BA plaques were evaluated using the Referenced measurement method

The results for the Referenced measurement reproducibility are presented in Table 4. The repeated measurements of the Referenced method were 8.569 ± 4.444 mm^2^ and 8.783 ± 4.892 mm^2^ for PA, 0.686 ± 0.185 and 0.684 ± 0.186 for SR, and 0.361 ± 0.112 and 0.361 ± 0.117 for PPB. No statistically significant differences were observed for those measurements (p-values > 0.05). The results indicate an excellent reproducibility for the repeated measurements, with the ICC and CV values ranging from 0.928 to 0.971 and 4.64 to 9.87%, respectively. Excellent agreement was also observed in the repeated measurement reproducibility analysis employing Bland-Altman plots (Fig. 5).

**Table 4:** Reproducibility of Referenced measurement method in assessing basilar plaque

	Measurement 1 (Mean ± SD)	Measurement 2 (Mean ± SD)	ICC (95% CI)	CV (%)	*p*
Plaque area (mm^2^)	8.569 ± 4.444	8.783 ± 4.892	0.971 (0.944–0.985)	9.87	0.289
Stenosis rate	0.686 ± 0.185	0.684 ± 0.186	0.970 (0.942–0.985)	4.64	0.774
Percent plaque burden	0.361 ± 0.112	0.361 ± 0.117	0.941 (0.887–0.969)	7.64	0.967

*SD* Standard deviation, *ICC* Intra-class coefficient, *CI* Confidence interval, *CV* Coefficient of variability

## Discussion

The present study evaluated the Self-referenced and Referenced measurement methods in assessing basilar atherosclerotic plaque employing dark blood HRMRI at 3 Tesla. The results show no significant differences between the Self-referenced and Referenced measurement. In addition, repeated measurements of PA, SR and PPB demonstrate excellent reproducibility in evaluating basilar atherosclerotic plaque.

This study employed Self-referenced and Referenced measurement methods to assess basilar atherosclerotic plaque and compared both techniques in evaluating morphologic parameters of the basilar artery. For the Self-referenced measurement method, the MLN site was used as the reference site to assess several parameters such as PA, SR and PPB. It is a simple and fast method to evaluate those parameters compared with the Referenced measurement method, in which both the nearest normal segments that are proximal and distal to the MLN site were used as the reference. Our data indicate that there were no significant difference between both methods in assessing the PA, SR and PPB of basilar plaque. In addition, only a small degree of bias was observed between the Self-referenced and Referenced measurement methods using Bland-Altman plots (Fig. 3). In addition, there was excellent reproducibility in the measurements of PA, SR and PPB by both methods, with ICC and CV values ranging from 0.941 to 0.990 and 3.73 to 9.87%, respectively. The reliability of these methods is of significant importance for the evaluation of basilar atherosclerotic plaque morphologic parameters. Briefly, the Self-referenced measurement method is a suitable alternative to the Referenced measurement method in quantifying basilar plaque morphology.

Dark blood HRMRI has been increasingly used to evaluate the morphology of basilar atherosclerotic plaque, as it offers good boundary outlines for the vessel-blood and the vessel-cerebral spine fluid borders [25, 31–33]. Kim et al. found that evaluation of stenosis associated with basilar atherosclerotic plaque using HRMRI is more accurate than evaluation using magnetic resonance angiography. The study involved a large sample size (219 patients) compared with other studies, and the Self-referenced measurement method was used to assess the degree of stenosis [23]. The discrepancy between HRMRI and magnetic resonance angiography was more obvious with mild stenosis, which shows that HRMRI is more sensitive for evaluating the early phase of intracranial atherosclerosis [23]. Ma et al. investigated the morphology of advanced basilar atherosclerotic plaque using the Referenced measurement method [26], Zhu et al. also used this method to assess the morphologic characteristics of atherosclerotic middle cerebral arteries using HRMRI at 3 Tesla [24] and Feng et al. explored arterial remodelling of basilar atherosclerosis in pontine infarction [25]. However, sites that were proximal and distal to the MLN were first selected, and then PA, SR and PPB were evaluated using the Referenced measurement method in those studies. In contrast, the Self-referenced measurement method, only needed the MLN site to calculate those parameters. In short, the main differences between the Self-referenced measurement method and the Referenced measurement method are presented as follows: (1) Only one site (MLN) was used in Self-referenced measurement method, while three sites (proximal, MLN and distal) were needed in the Referenced measurement method. (2) For plaque area quantification, the former method is more simple and rapid, as there is no need to calculate the reference outer wall, lumen and wall areas, which are needed in the latter method. Therefore, the Self-referenced method is a simple and convenient method for assessing plaque morphologic characteristics compared with the Referenced method. Our comparison of the Self-referenced and Referenced measurement method in evaluating basilar atherosclerotic plaque morphology demonstrates an excellent agreement between both the methods in assessment of PA, SR and PPB parameters, which indicates that the Self-referenced measurement method can be used in the clinical setting.

Imaging modalities including transcranial doppler ultrasound, computed tomography angiography and magnetic resonance angiography have been used to assess intracranial atherosclerosis. However, while those imaging techniques offer an evaluation of the vascular lumen, they are incapable of providing vessel wall information [23]. More importantly, the vessel wall characteristics can help us better understand the pathophysiology of atherosclerosis, which has a significant effect on patient management [4]. Dark blood HRMRI can delicately delineate plaque presence and morphology and provide new insights into atherosclerotic burden [2]. To increase the black blood effect, saturation band, inversion recovery, motion-sensitized driven-equilibrium, and delay alternating with nutation for tailored excitation are the most commonly used techniques during the magnetization preparation phase [34–37]. Because of the inherent flow void effect of the TSE protocol and due to its simplicity and low specific absorption ratio properties, the saturation band was used to saturate the inflow blood signal when imaging basilar plaque. Black blood high-resolution T2W images were used to obtain quantitative measurements because of the good contrast that was obtained between the lumen and plaque, the vessel wall and cerebrospinal fluid compared to the other imaging protocols [38].

The present study has several limitations. First, the sample size is relatively small. Further studies with a larger sample size are needed to validate the present results. Second, the Self-referenced measurement method could not be directly used to calculate the remodelling patterns of basilar atherosclerotic plaque, such as positive and negative remodelling patterns. However, we can use the Referenced measurement method to calculate those patterns if needed. Lastly, the outer wall, inner wall and lumen boundaries on the T2W images were manually outlined to assess the PA, SR and PPB of basilar atherosclerotic plaque. An automatic plaque measurement tool is needed, as this may improve efficiency and reduce errors between the repeated measurements.

## Conclusions

In conclusion, the Self-referenced and Referenced methods are slightly equivalent and both reliable, however, the former is quicker and easier and it may serve as a promising method for evaluation of basilar atherosclerotic plaque.

## Abbreviations

3D TOF-MRA: Three-dimensional time-of-flight magnetic resonance angiography
BA: Basilar artery
CV: Coefficient of variation
FOV: Field of view
HRMRI: High-resolution magnetic resonance imaging
ICC: Intraclass correlation coefficient
MLN: Maximal lumen narrowing
PA: Plaque area
PPB: Percent plaque burden
SD: Standard deviation
SR: Stenosis rate
TA: Acquisition time
TIA: Transient ischaemic attack
TR/TE: Repetition time/echo time
